# TEC disturbances caused by CME-triggered geomagnetic storm of September 6–9, 2017

**DOI:** 10.1016/j.heliyon.2024.e30725

**Published:** 2024-05-08

**Authors:** Chali Idosa Uga, Sujan Prasad Gautam, Ephirem Beshir Seba

**Affiliations:** a Jimma University, College of Natural and Computational Science, Department of Physics, Jimma, Ethiopia; b University of Alabama in Huntsville, Department of Space Science, Huntsville, AL, United States; c Ethiopian Space Science and Geospatial Institute, Department of Space Science and Research Application, Addis Ababa, Ethiopia; d KU Leuven, Department Wiskunde, Centre for Mathematical Plasma-Astrophysics, Leuven, Belgium^1^

**Keywords:** Geomagnetic Storm, Solar Activity, Coronal Mass Ejection, Ionospheric Disturbances, Total Electron Content

## Abstract

This study investigates the ionospheric response to a geomagnetic storm triggered by a Coronal Mass Ejection (CME) during 6–9 September 2017, across GPS stations located in diverse geographical regions. We analyze the changes in the magnetic field component (Δ*H*), the Prompt Penetration Electric Fields (PPEF), and the Total Electron Content (TEC). We find that Δ*H* exhibits latitude-dependent responses during the storm, with high-latitude stations experiencing more significant reductions compared to low-latitude stations. The PPEF behavior is found to be directly correlated with solar wind disturbances. Particularly during the main phase of the storm, fluctuations in PPEF were clearly associated with negative values in the Dst index. The KIRU station, located at a high latitude, shows the most pronounced PPEF effects, indicating the increased susceptibility of high-latitude regions to solar wind interactions. The time series plot of TEC, covering a full month at different stations, shows a distinct diurnal pattern driven by solar ionization. Equatorial stations such as HYDE, BOU, HON (HNLC), and DODM exhibit the highest daily TEC values. During the geomagnetic storm, TEC disturbances are evident across all stations, with significant disturbances and varying trends in TEC depletion rate observed at different locations. The TEC values differ by 5–25 TECU during the storm period, suggesting intricate ionospheric responses to geomagnetic storms at different stations. This highlights the importance of considering different geographical regions to fully understand the ionospheric dynamics related to solar activities.

## 1 Introduction

The disruptions in Earth's magnetosphere, due to its interaction with the CME-induced solar wind, are the principal source of large geomagnetic storms [1], [2] and significantly impact Earth [3]. The CME-induced solar wind contains a higher density of charged particles compared to typical solar wind. These particles can penetrate deeper into Earth's magnetosphere and subsequently interact with the ionosphere [4]. When these high-energy particles reach the ionosphere, they can collide with the neutral gases present there (such as oxygen and nitrogen). These collisions cause the neutral gases to become ionized, creating additional ions and free electrons in the ionosphere [5]. The increased ionization in the ionosphere during a CME-induced geomagnetic storm leads to a higher density of free electrons and ions, resulting in a change in the TEC distribution [6]. This is particularly noticeable in the F region of the ionosphere.

The ionospheric response to CMEs can vary, depending on the properties of the CME and the geomagnetic conditions. Some events may cause a positive ionospheric storm effect, where the TEC increases and then decreases during the main and recovery phases [7]. Other may have a negative ionospheric storm effect, where the TEC decreases during the main phase and recovers during the recovery phase [7]. The redistribution of ionospheric plasma during a geomagnetic storm can lead to significant disruptions in radio communications. These disruptions can impact the reliability and quality of signal propagation through the ionosphere, affecting various communication systems that rely on this medium [3], [8]. High-frequency (HF) radio signals can be absorbed more strongly in the ionosphere during such disturbances, affecting long-distance radio communications by changing in the path and velocity of radio waves [9], [10]. Additionally, radio signals can experience greater signal fading and signal path fluctuations as they pass through the fluctuating ionospheric regions [11]. CME-induced ionospheric disturbances can also affect satellite-based navigation systems like GPS (Global Positioning System) [12]. The radio signals from GPS satellites passing through the disturbed ionosphere may experience delays and errors in signal propagation [13]. These delays can lead to inaccuracies in GPS position estimates, affecting navigation and timing applications.

In the past, ionospheric TEC response to 7-8 September 2017 solar event has been widely studied. Different ionospheric responses have been observed in the different sectors of the globe. [14] examined the impacts of this event on the ionosphere, GNSS, and HF radio wave propagation at low and high-latitude regions. They found an abrupt rise of 8-10 TECU at mid-latitudes and 15-16 TECU enhancement at low-latitude regions. [15] examined ionosphere response during this event and found that in the Northern Hemisphere, there was a noticeable positive ionospheric effect, which amounted to an increase of approximately 65%, which is significantly higher. On the other hand, in the Southern Hemisphere, the ionosphere remained within its typical variation range, which is usually around ±40% during quiet times. Additionally, they observed different ionospheric responses over the same mid-latitude location in the Northern Hemisphere. Similar to their findings, [16] noted that there were more ionospheric irregularities in the high-latitude regions of the southern hemisphere compared to those in the high-latitude regions of the northern hemisphere. [17] studied ionospheric disturbances during this event over Asia, Africa, and the Americas. The findings showed a positive storm effect in Asia, a negative effect in Africa, and both effects in the Americas during the storm's main phase. [18] observed significant change in TEC by about 75% at low latitude stations of Hong-Kong region. [19] found significant increases in vertical total electron content over equatorial stations during this event. They observed a positive storm effect with a maximum dVTEC% of 16.7% followed by a negative ionospheric storm with dVTEC% of 32.8% took place during the first main phase and recovery phase. Additionally, a long-lasting positive ionospheric storm (∼10 hours) with a maximum dVTEC% of 26.3% occurred during the second main phase. [20] obtained prominent TEC enhancements at the crests of the equatorial ionization anomaly during the main phase of the storm. During the recovery phase on September 9, these TEC enhancements shifted to lower-latitude regions. Additionally, a moderate storm occurring before the strong storm on September 7 caused similar TEC enhancements at middle latitudes. However, during September 9-10, weak TEC depletion rates were observed at middle and low latitudes. Other several studies [21], [22], [23], [24], [25], [26], [27], [28] also examined ionospheric disturbances during this storm event over different regions. To date, open questions in understanding the main features of ionospheric disturbances still remain unsolved; so, monitoring the TEC variations over different sectors during CME events is important for assessing potential space weather impacts.

A prompt penetrating electric field (PPEF) during geomagnetic storms is also commonly studied [29], [30], [31]. It is an electric field that rapidly penetrates the Earth's ionosphere during a geomagnetic storm. These electric fields are driven by the leakage of high-latitude electric fields and can have significant effects on the ionosphere and the Earth's magnetic field [29], [32]. During a geomagnetic storm, wind-driven currents in the ionosphere, coupled with the Earth's magnetic field, produce the Equatorial Electric Field (EEF), which is responsible for driving PPEFs [33]. PPEFs, along with other ionospheric disturbances such as the Disturbance Dynamo Electric Field (DDEF), can lead to the intensification of the Equatorial Ionization Anomaly (EIA). The EIA is a region of enhanced ionization near the equator, and its intensification can be attributed to the effects of PPEFs [34]. In addition, it can generate convection electric fields, which can reach up to 5.1 mV/m at low latitudes; these electric fields are strong enough to cause ionospheric disturbances, such as the enhancement of the TEC and the EIA [35]. Furthermore, PPEFs also drive the formation of Hall and Pedersen current circuits, which are responsible for middle latitude geomagnetic disturbances [36]. These disturbances can further affect the ionosphere and the Earth's magnetic field.

Geomagnetic storms have also a significant impact on geomagnetic field at different sectors [37]. These impacts occur in multiple ways, primarily through fluctuations in the strength and orientation of Earth's magnetic field [38]. Magnetometer measurements reflect dynamic processes, including fluctuations in magnetic field strength and the phenomenon of sudden commencements (SCs) that signal the storm's onset [39]. Moreover, geomagnetic pulsations, which are periodic oscillations in the magnetic field, often arise due to resonant interactions within the magnetosphere and the interplay between the solar wind and Earth's magnetic field [40]. These fluctuations in the magnetic field continue throughout the main phase of the storm. Importantly, the influence of geomagnetic storms on the geomagnetic field can vary depending on the geographic location of a magnetometer station, with latitude variances playing a significant role [41]. Following the recovery of the storm, magnetometer data gradually return to their pre-storm condition as the Earth's magnetosphere reverts to its normal configuration [40]. The observations and analyses of components of the magnetic field during the geomagnetic storm are also crucial for weather monitoring and assessing the impact of geomagnetic storms on various technological systems [42], [37], [14].

Although investigations on TEC responses due to the 6-9 September 2017 geomagnetic storm event have been widely performed, the aforementioned literature confirmed that the TEC at different stations exhibits distinct responses to space weather events and the ionospheric effects can vary even at the shorter distanced stations. The study of those regional variations is crucial for refining models and predictions of ionospheric behavior. In addition, there is a notable gap in the literature concerning the response of the component of Earth's magnetic field and PPEFs, particularly at the considered stations during this event. Thus, in this work, we focus on an analysis of the ionospheric TEC and geomagnetic field responses to this storm event at rarely-studied high, middle, and low-latitude stations. Our paper is organized as follows. In section 2, we discuss the data and methods used. We present our results in section 3. In section 4, we discuss our results. Finally, we conclude our results in section 5.

## 2 Data and methods

**Figure 1:**
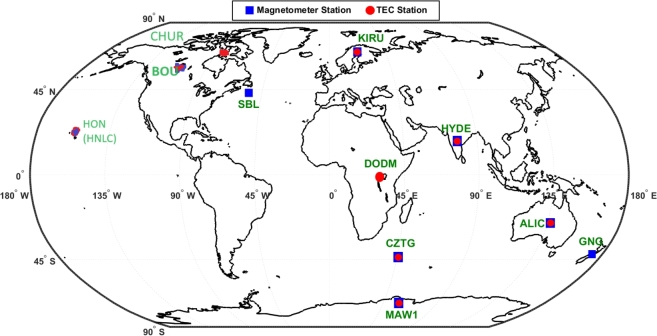
Location of the selected stations on the global map.

In this work, to analyze the effect of geomagnetic storm of 6-9 September 2017, we use 1-hour resolution data of solar wind parameters and geomagnetic indices, and 1-minute resolution data of ionospheric TEC and component of Earth's magnetic field. We download data from multiple sources. The TEC data are obtained from the GAGE Data Server (https://gage-data.earthscope.org/archive/gnss/rinex). The data from this source is originally in the RINEX format; thus, to calibrate these data, we process them through the GPS-TEC analysis program [43], [44]. TEC responses to the geomagnetic storms differ largely according to the geographic locations [45]. These days, researchers perform global tracking of GPS-TEC using various models such as IGS Global TEC map [46], [47], [45]. Although these models provide good signature of how TEC is varying over the different parts of the globe during the geomagnetic storms, the non-uniform accuracy of IGS TEC data in the global map may have a restriction on the accuracy of the TEC [48]. Thus, in order to obtain a thorough understanding of how various latitudinal regions react to geomagnetic disturbances and impact on communication, navigation, and other technological systems, we use total 9 GPS stations at the different sectors: low-latitude stations (HYDE and DODM, HNLC), mid-latitude stations (ALIC, BOU, CHUR, SBL, and GNG), and high-latitude stations (KIRU, CZTG, and MAW1). The detailed information of all stations including geographic latitude and longitude is given in Table 1. The locations of the stations are also displayed on the global map in Fig. 1. To observe the geophysical conditions, we use solar wind parameters, including the z-component of interplanetary magnetic field (IMF) Bz, interplanetary electric field (IEF) Ey, plasma speed (Vsw), and geomagnetic indices data, such as the Auroral Electrojet (AE-index), Kp, and Dst, during the studied period. We download these data sets from the Operating Mission as Nodes on the Internet Web (OMNI) system (https://omniweb.gsfc.nasa.gov/).

**Table 1:** Geographical information of the selected stations.

Code	Station Name	Geog. lat	Geog. long	Geomag. lat	Geomag. Long	Data Type	Local Time
ALIC	Alice Springs	23.67^*o*^S	133.88^*o*^E	34.35^*o*^S	153.07^*o*^W	TEC	UT+9:00
						Magneto	
MAW1	Mawson	67.67^*o*^S	62.87^*o*^E	70.13^*o*^S	89.92^*o*^E	TEC	UT+4:00
						Magneto	
CZTG	Crozet Island	46.46^*o*^S	51.85^*o*^E	53.20^*o*^S	106.07^*o*^E	TEC	UT+3:00
						Magneto	
DODM	Dodoma	6.18^*o*^S	35.74^*o*^E	16.32^*o*^S	107.07^*o*^E	TEC	UT+2:00
GNG	Gingin	31.36^*o*^S	115.72^*o*^E	43.96^*o*^S	173.68^*o*^W	Magneto.	UT+9:00
HNLC	Honolulu	21.30^*o*^N	157.87^*o*^W	21.33^*o*^N	89.81^*o*^S	TEC	UT-11:00
						Magneto	
BOU	Colorado	40.05^*o*^N	105.16^*o*^W	34.80^*o*^N	39.79^*o*^W	TEC	UT-7:00
						Magneto	
CHUR	Churchill	58.76^*o*^N	94.08^*o*^W	68.64^*o*^N	27.02^*o*^W	TEC	UT-6:00
KIRU	Kiruna	67.9^*o*^N	20.9^*o*^E	64.29^*o*^N	105.06^*o*^E	TEC	UT+1:00
						Magneto	
HYDE	Hyderabad	17.41^*o*^N	78.55^*o*^E	9.62^*o*^N	150.7^*o*^E	TEC	UT+5:00
						Magneto	
SBL	Sable Island	43.93^*o*^N	299.99^*o*^E	53.57^*o*^N	21.60^*o*^E	Magneto.	UT+5:00

We calculate dTEC (TEC deviation) to observe the change TEC during the storm period (storm-TEC) in comparison to TEC during the quiet period (quiet-TEC). dTEC is commonly used to examine the differences between two sets of data. Here, it quantifies how far the storm-TEC deviate from the quiet-TEC. A lower dTEC indicates that the storm-TEC and quiet-TEC are more similar or have a smaller overall deviation, while a higher dTEC suggests greater dissimilarity or a larger overall deviation. dTEC can be estimated using the relation,

(1) $$dTEC=\frac{TEC_{s}-TEC_{q}}{TEC_{q}}$$

where $TEC_{s}$ is the TEC during the storm period (September 6–9) and $TEC_{q}$ is the median of the TEC during the quiet days of the September month. Note that, we just take geomagnetically undisturbed days for the estimation of quiet days. Other storm days (analyzing Dst, Ap and Kp indices) are not discarded. We also confirm our quiet days from the International Q-Days and D-Days of GFZ, Potsdam (https://wdc.kugi.kyoto-u.ac.jp/qddays/index.html).

We also study a variation of PPEF during the storm and quiet period using Promt Penetration Electric Field Model (PPEFM). It is a transfer function model that predicts daily fluctuations in the solar wind that are mapped in IEF data [49]. The transfer function simulates current equatorial ionosphere fluctuations using real-time data from the ACE satellite. The model uses time and longitude of the station as input factors to obtain the best estimations of the equatorial electric field. The model outputs show the electric field produced by the convective electric field, the quiet time electric field, and a combination of the two [50], [51], [52]. We use the Ionospheric Electric Fields Real-time Model data obtained from the website (https://geomag.colorado.edu/real-time-model-of-the-ionospheric-electric-fields).

Furthermore, we use magnetometer data to observe the variation in the horizontal component of Earth's magnetic field at each considered station. The magnetic field data are taken from the International Real-Time Magnetic Observatory Network (INTER MAGNET) (https://intermagnet.org/). This site offers one-minute resolution data for the northward (*X*), eastward (*Y*), and vertical (*Z*) components of the Earth's magnetic field, while the horizontal component (*H*) can be calculated using Equation (2) [53], [54] as,

(2) $$H=\sqrt{X^{2}+Y^{2}}$$

To avoid different offset values of different magnetometers, control of the reference frame, scale factors, and temperature effects of the magnetometer, the night time baseline values in the *H* component are first obtained using Equation (3) [55] for each day and subtract from the corresponding magnetometer data sets to obtain the hourly departure of *H* given by the Equation (4). The baseline value is defined as the average of the night time (23:00-02:00 LT) *H* component value of the Earth's magnetic field.

(3) $$H_{o}=\frac{H_{23}+H_{24}+H_{01}+H_{02}}{4}$$

Where $H_{23}+H_{24}+H_{01}+H_{02}$ are the summation of the hourly values of H at 23:00, 24:00, 01:00 and 02:00 in local time (LT), respectively.

(4) $$\Delta H=H(t)-H_{o}$$

where *t* is the time in hours ranging from 01:00 to 24:00 LT.

## 3 Results and discussion

### 3.1 Geophysical conditions of the September 6-9, 2017 event

**Figure 2:**
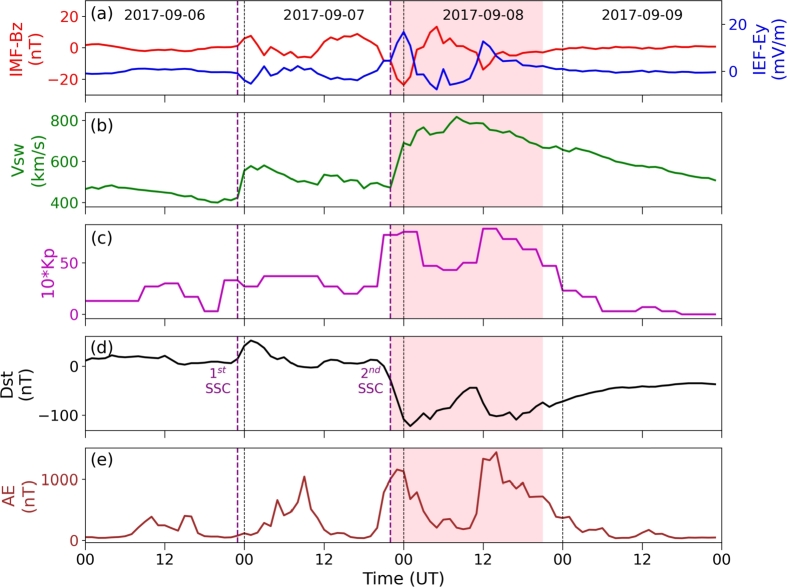
Variation on solar wind parameters and geomagnetic indices during the storm period of 6-9 September 2017. The top-to-bottom panels labeled from (a) to (e) represent IMF-Bz and IEF Ey, Vsw, Kp-index, Dst index, and AE index, respectively.

The 7-8 September 2017 solar event was characterized by a series of solar flares and geomagnetic storms, with the X9.3 flare on September 6, 2017, being the most intense flare recorded during the solar cycle (for more detailed description on these flares, see [56]). The CME originating from the powerful X9.3 solar flare on September 6th reached Earth at 23:00 UT on September 7th [57]. Its arrival caused a significant disturbance in the Earth's magnetosphere, resulting in a severe geomagnetic storm with two main phases of great intensity separated by about 14 hours and the highest recorded geomagnetic index value of Kp = 8 [57], [58]. Fig. 2(a-e) depicts an overview of the temporal variation of the solar wind parameters and space weather indices during the period of 6-9 September 2017. These parameters include IMF-Bz, IEF-Ey, Vsw, Kp index, Dst index, and AE index. Purple dashed lines represent the 1st and 2nd Sudden Storm Commencement (SSC) occurred. The shaded region represents the storm-effective period. On September 8th, 2017, a G4 category storm occurred. The interplanetary shock on September 7th, around 23:00 UT, begin the storm's initial phase, characterized by rapid changes in the mentioned parameters. During the main phase, the Bz component of the IMF is pointed southward, reaching its lowest value ∼-24 nT at 00:00 UT, before quickly rising to around ∼+14 nT. It is shifted southward again, reaching a negative value of ∼-14 nT at around 12:00 UT, and remained southward until around 2:00 UT. Then, the Bz component gets gradually decreased and remains around 0 nT from September 9th. On September 8th, a significant change in solar wind speed is occurred, peaking at around ∼820 km s^−1^ around 08:00 UT and gradually decreasing after 12:00 UT. The IMF-Ey is also showing notable fluctuations during the storm, influenced by the Bz component of the IMF and the X component of the Vsw, ranging between -15 and +20 mV/m during the storm. During the main phase of the storm, the Dst-index is decreased, reaching a negative value at around 01:00 UT, marking the first minimum of ∼-122 nT. In the partial recovery phase from 01:00 to 11:00 UT, it is increased from ∼-122 nT to ∼-44 nT. It decreases again, reaching its second minimum of ∼-110 nT, ending the main phase, lasting for approximately 14 hours. The main phase is characterized by two significant minima in the Dst-index, occurring at around 01:00 and 14:00 UT on September 8th, 2017. The recovery phase begins after around 02:00 UT on September 8th, and the SYM-H index is gradually increased, returning to its normal value from September 9. The Kp index shows two distinct maxima, peaking its value at about +8 between 00:00–03:00 and 12:00–15:00 UT on September 8th. In the AE index, multiple peaks are evident during this period. Following the arrival of the first CME, there is a notable increase in auroral activity, with the AE index reaching its highest point at approximately ∼1158 nT on September 7th at 09:07 UT. However, the presence of two significant peaks with AE values exceeding ∼1442 nT indicates that the most intense aurora activity is occurred following the arrival of the second CME.

### 3.2 Variations of geomagnetic field component

**Figure 3:**
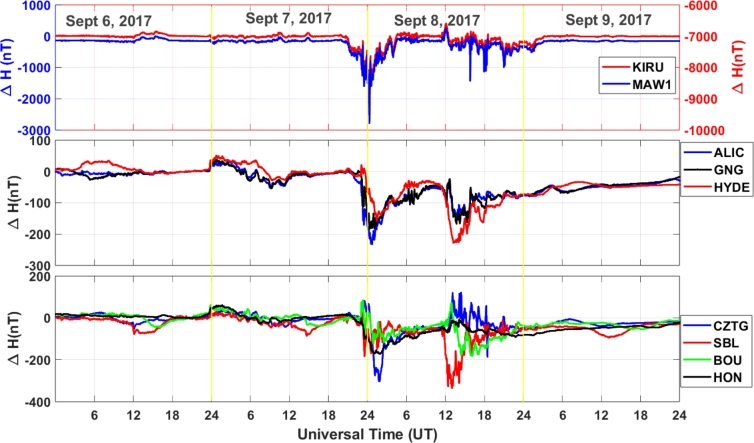
Variations of Δ*H* at different stations during September 6-9, 2017 geomagnetic storm.

Fig. 3 shows the variations of Δ*H*, which is calculated using Equation (4), for seven different stations before, during, and after the geomagnetic storm of September 6-9, 2017. During the geomagnetic storm, we observe a notable change in Δ*H* at all monitoring stations. However, the depletion of Δ*H* values is more pronounced at the high-latitude stations (KIRU and MAW1) compared to the low- and mid-latitude stations. This clearly indicates that the Δ*H* response during the geomagnetic storms varies according to the longitude and latitude. This is due to the fact that the magnetic field varies in strength over the earth's surface, being greatest near the poles and weakest towards the equator [59], [60], [61]. Figure shows that Δ*H* reach to less than -2000 nT at KIRU station and around -1000 nT at MAW1 station. In this event, the KIRU station located in the northern hemisphere is experiencing a larger depression in Δ*H* in comparison to the MAW1 station situated in the southern hemisphere. At the low- and mid-latitude stations, ALIC, HYDE, GNG, BOU, and HON, Δ*H* has a similar trend with values around -100 to -200 nT, and at the stations SBL and CZTG, the Δ*H* value is decreased to approximately -300 nT during the geomagnetic storm. During the first phase of the storm, the CZTG station exhibits a more substantial reduction in Δ*H* compared to the SBL station. However, during the second main phase of the storm, while the SBL station displays a significant decrease in Δ*H*, the CZTG station experience fluctuations around the 0 nT value. The variations in Δ*H* that occur in low- and middle-latitude regions during the early stages of a geomagnetic storm are mostly caused by increased ionospheric currents that are brought about by magnetospheric compression and substorm activity [62], [63]. On the other hand, large-scale geomagnetic field disruptions linked to magnetospheric convection and auroral activity, as well as strong auroral electrojets, are the major causes of the larger depressions seen during the storm's main phase in high-latitude locations [64], [65]. In general, it is important to note that the exact behavior of geomagnetic storms can vary depending on the strength and direction of the solar wind and the specific characteristics of each storm event and both the Northern and Southern Hemispheres can experience significant changes in the magnetic field.

**Figure 4:**
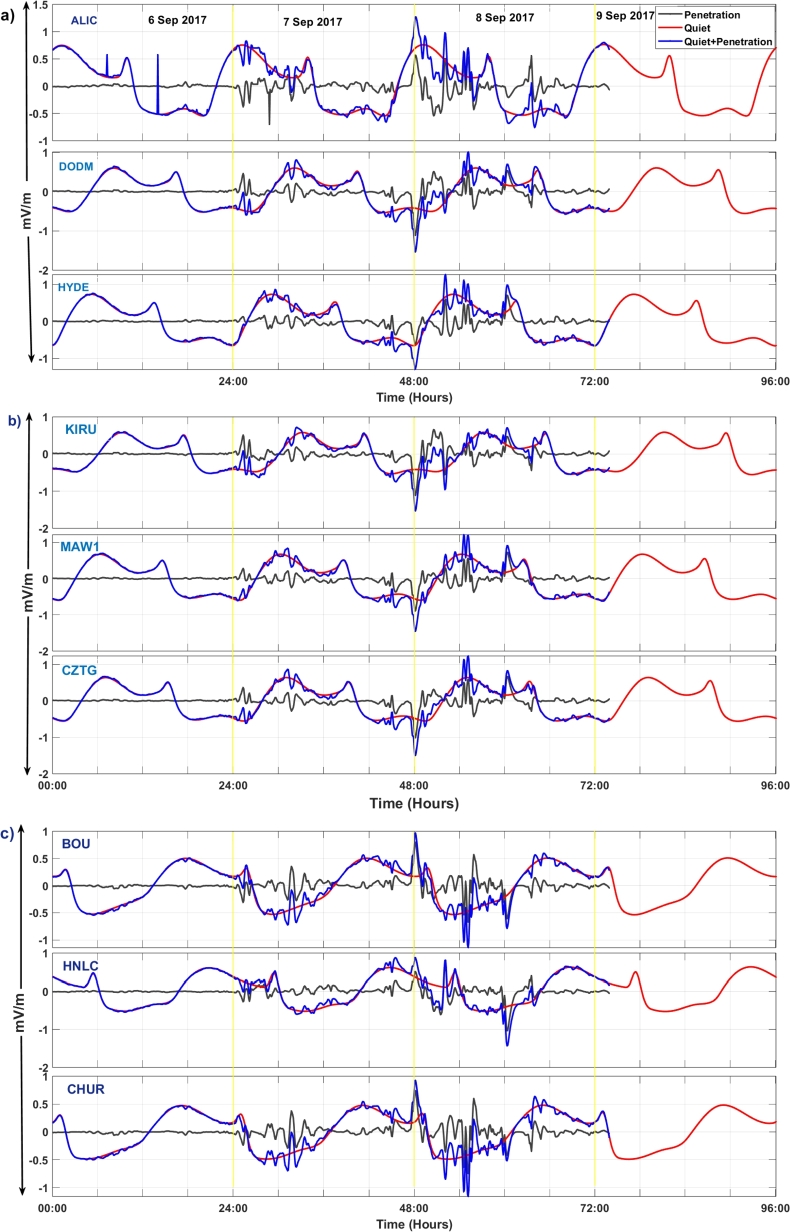
Variations of prompt penetration electric fields (PPEF) during September 6–9, 2017 over all stations: Panel a) indicates PPEF variations over ALIC, DODM, and HYDE. Panel b) indicates variations over KIRU, MAW1, and CZTG stations, and finally, Panel c) indicates PPEF variations over BOU, HNLC, and CHUR stations. Their y-label indicates the units of prompt penetration electric fields.

Fig. 4 (a-c) depicts the variations of the PPEF using the quiet-time electric field and the penetration electric fields before and during the geomagnetic storm activity over the suggested stations. On September 6, there is no variation on penetration, quiet time electric field and quiet time plus (+) penetration electric field. The fluctuations of the solar wind interplanetary electric field and the penetration electric field start during the initial phase of the storm and fluctuate more during the main phase of the storm. When the Dst index and the horizontal components of the Earth's magnetic field become more negative, especially between 00:00 UT and 02:00 UT on September 8, 2017, the ionospheric prompt penetration electric fields are suppressed over each station. KIRU station shows higher fluctuations in comparison to the other stations, indicating a higher PPEF effect over northern hemispheric high-latitude stations. High-latitude stations are located at higher geomagnetic latitudes, meaning they are closer to the Earth's magnetic poles. This proximity makes them more susceptible to the direct effects of solar wind disturbances and magnetic field variations associated with geomagnetic storms. In these regions, the Earth's magnetic field lines are more vertically oriented. When the solar wind interacts with the Earth's magnetic field, it may have a more direct and pronounced effect on high-latitude regions. This may lead to more significant variations in electric fields. [21] mentioned that PPE field and composition caused TEC enhancement in low latitude region and it was absent in the equatorial region. These results are also supported by [52] and [26]. The combined impacts of IMF orientation, geomagnetic latitude dependency, and ionospheric response characteristics may cause the values of PPEF to decline more over high latitude stations during geomagnetic storms than over low and middle-latitude stations [66], [67]. However, the particulars of each geomagnetic storm occurrence and the locations of monitoring sites might affect the distinctive behavior of PPEF. We note that the presence of PPEF has been observed through other various methods beyond just this model outputs from magnetic field observations. One method involves utilizing automated learning and machine learning models to predict PPEF values accurately. For instance, Gaussian Process Regression (GPR) models have been developed to estimate PPEF values with high accuracy, outperforming other models like Adaptive Neuro-Fuzzy Inference System (ANFIS) and Artificial Neural Network (ANN) [68]. These models also have been applied to predict PPEF values in datasets collected from different regions, enhancing the understanding of these electric fields. The results from different models can be compared.

### 3.3 Variability of ionospheric TEC during September 1-30, 2017

**Figure 5:**
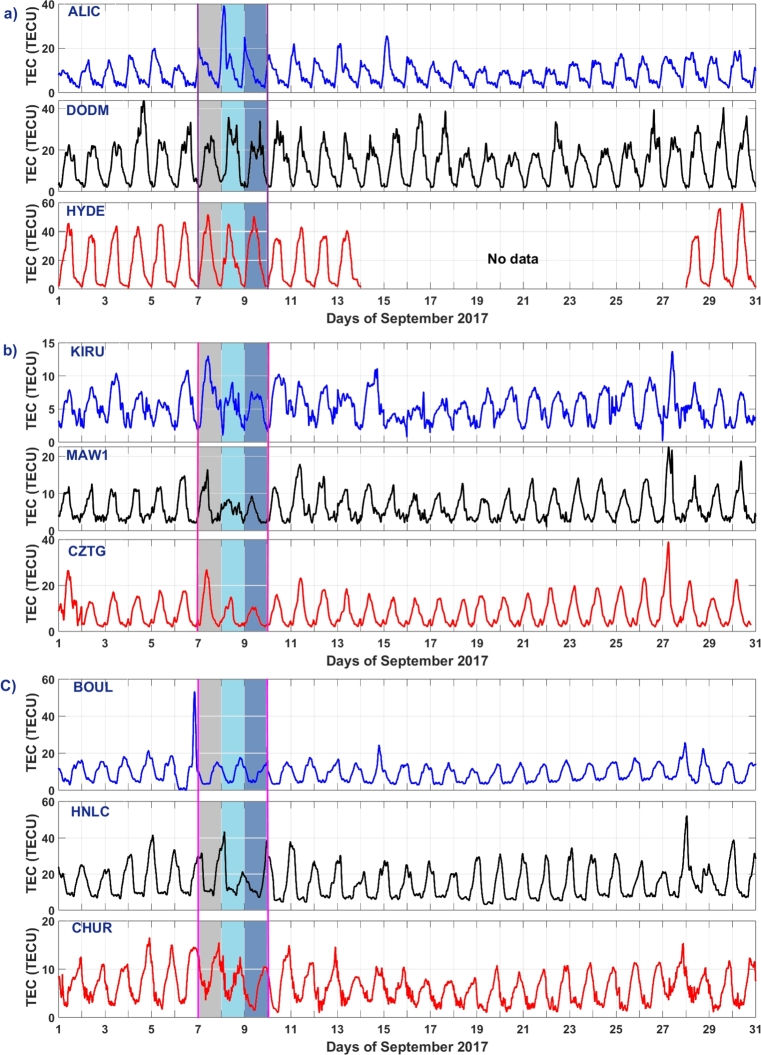
Time series plot of TEC in TECU at selected stations during the month of 2017. Panel a) indicates TEC over ALIC, DODM, and HYDE. Panel b) indicates TEC over KIRU, MAW1, and CZTG stations, and finally, Panel c) indicates TEC over BOU, HNLC, and CHUR stations. The shaded region indicates the storm-impacted days. Grey, light-blue, and dark-blue shaded regions indicate the days when the initial phase, main phase, and recovery phase, respectively, occurred during the geomagnetic storm.

Fig. 5 (a-c) shows the time series plot of TEC over the ALIC, DODM, HYDE, KIRU, MAW1, CZTG, BOUL, HON (HNLC), and CHUR stations spanning the period from September 1–30, 2017. The plots illustrate a clear diurnal (daily) pattern, with values typically rising during daylight hours and decreasing at night. This diurnal variation is driven by the Sun's influence on the ionosphere. Electron density increases during the day due to solar ionization and decreases at night when solar radiation diminishes. During the geomagnetic storm period, September 7-9, 2017, TEC disturbances are clearly visible over all stations. The diurnal maximum TEC value is found to fluctuate day to day. The greater value of daily TEC can be observed at the low latitude stations, HYDE and DODM. At these stations, TEC values are ranging from 20-50 TECU. At the mid-latitude station (ALIC), TEC values are between 10-20 TECU on average. Over BOU and HON (HNCL) stations, TEC values range from 54 to 58 TECU and from 15 TECU over the CHUR (Canada) sector. BOU and HON stations frequently exhibit abnormal features as compared to their other longitudinal analogs. Furthermore, those stations are more directly exposed to solar radiation and may exhibit higher TEC levels during periods of elevated solar activity. However, at high latitude stations, KIRU, MAW1, and CZTG, TEC values are ranging from 5-15 TECU. The combination of various factors such as the equatorial anomaly, solar zenith angle, electrojet currents, low geomagnetic activity, and favorable geophysical conditions leads to higher TEC values in equatorial regions. These regions are characterized by their unique ionospheric properties that result in greater electron density and TEC. During a period of low geomagnetic activity on September 4, the DODM station displayed a notable increase in TEC, a phenomenon not observed at other monitoring stations. Furthermore, upon closer examination of the daily TEC patterns, distinct variations were evident among the different stations. Diurnal TEC variations may vary over different regions due to other several factors such as differences in latitude, seasonal changes, and local environmental factors.

### 3.4 Variability of ionospheric TEC during September 6-9, 2017

**Figure 6:**
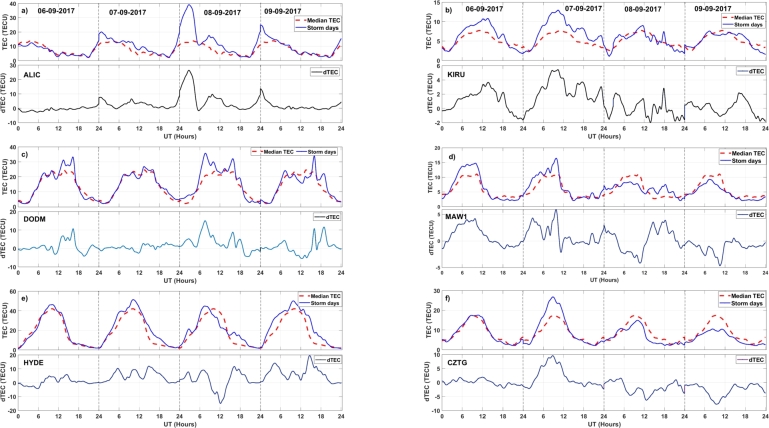
Variations of TEC during the normal days (dotted red line) and TEC during the event days (solid blue line) from September 6-9, 2017 with corresponding dTEC over different stations.

**Figure 7:**
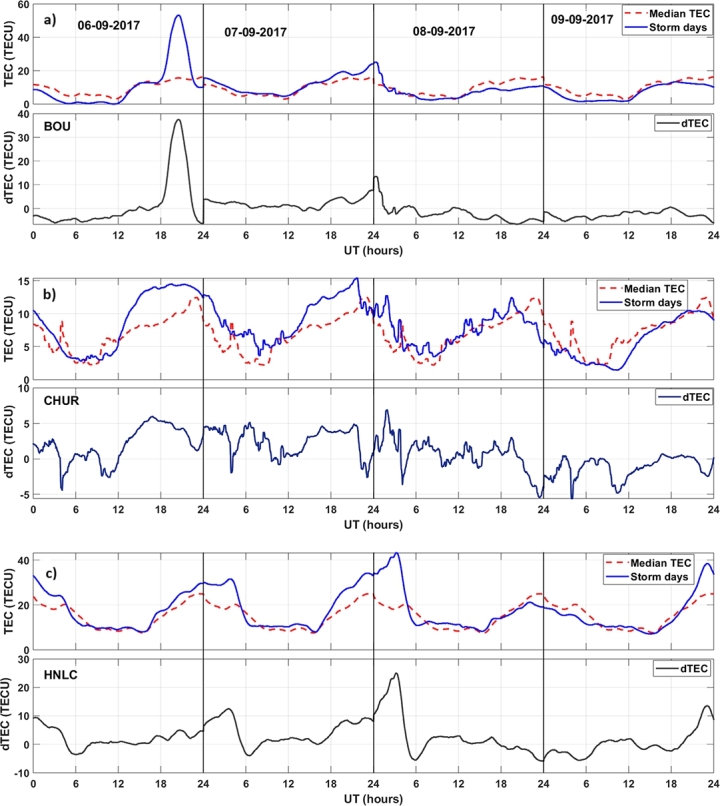
Variations of TEC during the normal days (dotted red line) and TEC during the event days (solid blue line) from September 6-9, 2017 with corresponding dTEC over BOU, HON (HNLC), and CHUR stations.

The dTEC is computed using Equation (1) to investigate variations in TEC between a disturbed day and normal days. The variations of ionospheric TEC at different stations during the space weather event of September 6-9, 2017 are presented in Figs. 6 (a-f) and 7 (a-c). The red dotted plot in the figure is the median TEC value of the quiet time period. The blue solid line represents the TEC values during the disturbed period. The figure illustrates significant disturbances in TEC during the storm period. However, it's noteworthy that we observe variable trend in TEC changes. At the ALIC station, a substantial positive change in TEC is evident during the storm, with the maximum dTEC reaching more than 35 TECU. Similarly, the DODM station exhibits a positive dTEC of approximately 15 TECU during the storm period. However, at the HYDE station, TEC is increased on September 7th, reaching a maximum dTEC value of approximately 12 TECU. These results are consistent with the results of [25] and [57] at mid and low-latitude stations. On September 8th, dTEC fluctuates between -12 and 10 TECU. The following day, dTEC remains positive throughout. At the CZTG station, TEC increases on September 7th and decreases on September 8th, with a maximum dTEC value of approximately 9 TECU and -6 TECU, respectively. The MAW1 station shows a dTEC value of around 9 TECU on September 7th. On September 8th, there is a decrease in dTEC of about -6 TECU until noon, followed by a positive increment of 4 TECU in the afternoon. On September 9th, dTEC remains below the median TEC for the entire day, a trend also is observed at the CZTG station. Additionally, at the KIRU station, dTEC is positive on September 6th and 7th, with values of approximately 3 TECU and 5 TECU, respectively. On September 8th, dTEC exhibits fluctuations ranging from -2 to 3 TECU. We observe higher fluctuations in TEC pattern in high latitude stations, this supports the results of [69]. Interestingly, at the BOUL station, a substantial positive change in TEC was evident during September 6, 2017 than during the storm days, with the maximum dTEC reaching more than 56 TECU. Similarly, the HON (HNLC) station exhibits a positive dTEC of approximately 25 TECU during the storm period. However, at the CHUR station, TEC starts increasing on September 7th, reaching a maximum dTEC value of approximately 7 TECU during the main phase of the storm. During geomagnetic disturbances, both Disturbance Dynamo Electric Field (DDEF) and PPEF generally contribute to TEC enhancements at low- and middle-latitude stations. However, the relative importance of each factor may differ based on the particulars of the storm event, the stations' geographic location, and other ionospheric conditions [70], [71].

## 4 Discussion

The results of this study show that during the geomagnetic storm of September 6-9, 2017, there is a notable change in Δ*H* at all monitoring stations. However, the suppression of Δ*H* values is more pronounced at the high-latitude stations compared to the low-latitude stations. Station situated at middle latitude exhibits a decrease in Δ*H* values that fall between the reductions observed at low-latitude and high-latitude stations. This clearly indicates that the Δ*H* response during the geomagnetic storms varies according to the longitude and latitude. This supports the previous study of [72]. This latitude-dependent behavior can be attributed to the Earth's varying magnetic field strength, which is strongest near the poles and weakest near the equator [73]. PPEFs are driven by the leakage of high-latitude convection electric fields to low latitudes. During geomagnetic storms, the convection electric fields are enhanced, resulting in stronger PPEFs [29]. Disturbance dynamo electric fields are driven by the storm-time neutral winds through the action of the ionospheric dynamo processes [32]. These electric fields can also contribute to the variation in PPEF during geomagnetic storms. We find maximum PPEF fluctuation at KIRU station, at high latitude station. This may be because, higher PPEF levels are often linked to a reduction in the Earth's magnetic field's horizontal component (H component), since PPEF disrupts ionospheric currents during geomagnetic storms. BOUL and ALIC stations, which are at middle latitude, also show significant fluctuations in comparison to other stations. High latitude regions are closer to the Earth's magnetic poles, where the geomagnetic field is stronger. This results in a larger variation in PPEF in these regions [35]. The convection electric fields that drive PPEFs are stronger at high latitudes, resulting in larger fluctuations in PPEF [29]. On comparing Figure 3, Figure 4, we see that dH and PPEF response to geomagnetic storm are identical. This suggests that the PPEF model is well-capturing the disruption on PPEF due to the storm. The diurnal variation of TEC is driven by the Sun's influence and other factors on the ionosphere. During the geomagnetic storm period, TEC disturbances are clearly identifiable over all stations. The greater value of daily TEC can be observed at the equatorial stations. At these stations, TEC values are ranging from 20-50 TECU. Middle latitude stations have TEC values between 10-58 TECU. However, at high latitude stations, TEC values are ranging from 5-15 TECU. Diurnal TEC variations may vary over different regions due to various reasons. The equatorial region receives more solar radiation compared to high latitude regions, which results in more ionization of the upper atmosphere and hence higher TEC values [74]. In addition, the equatorial anomaly is a region of the ionosphere where the TEC values are significantly higher than the surrounding regions. This is due to the convergence of the geomagnetic field lines at the magnetic equator, which results in a higher concentration of free electrons [75]. Furthermore, the plasma fountain effect is a process in which plasma is transported from the lower ionosphere to the upper ionosphere. This effect is more pronounced in the equatorial region, which results in higher TEC values [76]. Also, the polar cap absorption, a process in which energetic particles from the solar wind are absorbed by the ionosphere in the polar regions, results in a decrease in TEC values in these regions [77]. The variations of ionospheric TEC at different stations during the space weather event shows significant disturbances in TEC. However, it's noteworthy that there was a variable trend in TEC changes observed. The ALIC station shows larger dTEC during the main phase of the storm, which is not seen over other stations. This variation in TEC measurements can also be attributed to differences in the quality and precision of instruments at different stations. Factors like the altitude of the receiver, receiver sensitivity, and the processing techniques used also can impact TEC measurements. Further ionospheric analysis can be done by taking other various stations in the Australian region in order to understand this pronounced effect of the storm in that region. The TEC change is occurring earlier in high-latitude stations such as KIRU and MAW1. The convection electric fields that drive PPEFs are stronger at high latitudes, resulting in larger fluctuations in PPEF [78]. During geomagnetic storms, the convection electric fields are enhanced, resulting in stronger PPEFs. This effect is more pronounced at high latitudes, resulting in earlier changes in TEC compared to low and middle latitudes [79]. Further, the ionospheric composition varies with latitude, with different ion species dominating at different latitudes. During geomagnetic storms, the ionospheric composition can change, resulting in different responses in TEC at different latitudes [80]. Some regions may have local magnetic anomalies, such as magnetic anomalies associated with geological features. These anomalies also can affect how the geomagnetic storm impacts TEC in those areas.

We use magnetometers and GPS-TEC measurements at many locations spread across eleven nations to examine the variations in the ionospheric TEC; however, it does not explain the global trend of the ionosphere. Using various measurements, [45], [47] and other works in their references have established the global trend of ionospheric TEC. Similar map can be constructed for other future events using different models and measurements. In general, this work is crucial for understanding the TEC disruptions caused by geomagnetic storms, and it aids in space weather forecasting and mitigation initiatives. Future studies will focus on understanding the physical processes that cause TEC disruptions during geomagnetic storms, such as interactions between the solar wind and the magnetosphere, ionospheric plasma instabilities, and magnetosphere-ionosphere coupling processes. Additionally, ongoing modeling research and observational missions will help anticipate and mitigate the effects of geomagnetic storms on technological systems and refine space weather forecasting models.

## 5 Conclusion

In conclusion, our investigation of the ionospheric responses to the CME-triggered geomagnetic storm from September 6-9, 2017, provided some significant findings. The response of Δ*H*, the Earth's horizontal magnetic field component, during the storm exhibited a latitude-dependent pattern, with high-latitude stations experiencing more pronounced reductions in magnitude compared to those at lower latitudes. The varying strength of Earth's magnetic field across latitudes played an important role in shaping these responses. Analysis of PPEF showed their direct correlation with solar wind disturbances, evident through fluctuations in solar wind parameters during the storm period. Notably, the KIRU station at high latitudes showed the most notable PPEF fluctuation, indicating the heightened vulnerability of regions at higher latitudes to interactions with solar wind. The month-long time series plot of TEC illustrated a distinct diurnal pattern influenced by solar ionization, with daily TEC peaks varying day to day. Throughout the geomagnetic storm period, a noticeable increase in TEC was observed across various stations, accompanied by a significant change in TEC denoted by larger dTEC values. However, the dTEC values displayed variability across different stations, emphasizing the complex interplay of solar activity, geomagnetic field strength, ionospheric composition, and local geographical and magnetic factors in influencing TEC variations during geomagnetic storms. Finally, it is found that the local time at which the storm occurred plays a significant role in controlling the impact of storm on TEC at each location.

## Funding

There was no funding used for the publication of this paper.

## CRediT authorship contribution statement

**Chali Idosa Uga:** Writing – review & editing, Writing – original draft, Supervision, Software, Methodology, Investigation, Formal analysis, Data curation, Conceptualization. **Sujan Prasad Gautam:** Writing – review & editing, Writing – original draft, Software, Methodology, Investigation, Conceptualization. **Ephirem Beshir Seba:** Writing – review & editing, Visualization, Supervision, Software, Methodology, Conceptualization.

## Declaration of Competing Interest

The authors declare that they have no known competing financial interests or personal relationships that could have appeared to influence the work reported in this paper.

## Data Availability

Data will be available from the corresponding author upon request.
